# Use of Geopolymer and Carbon Fiber-Reinforced Polymer for Repairing Reinforced Concrete Deck Soffit

**DOI:** 10.3390/ma16124459

**Published:** 2023-06-19

**Authors:** Yeou-Fong Li, Guo-Wei Hao, Jin-Yuan Syu, Bian-Yu Chen, Wei-Hao Lee, Ying-Kuan Tsai

**Affiliations:** 1 Department of Civil Engineering, National Taipei University of Technology, Taipei 10608, Taiwan; yfli@mail.ntut.edu.tw (Y.-F.L.); jerrylovehnhn@gmail.com (G.-W.H.); t9679010@ntut.org.tw (J.-Y.S.); chen.ben968@gmail.com (B.-Y.C.); 2 Institute of Mineral Resources Engineering, National Taipei University of Technology, Taipei 10608, Taiwan; whlee@ntut.edu.tw; 3 Department of Environmental Information and Engineering, Chung Cheng Institute of Technology, National Defense University, Taoyuan 33550, Taiwan

**Keywords:** geopolymer, carbon fiber reinforcement, flexural strength, cyclic third-point loading test

## Abstract

This study aimed to assess the feasibility of utilizing geopolymer for repairing reinforced concrete beams. Three types of beam specimens were fabricated: benchmark specimens without any grooves, rectangular-grooved beams, and square-grooved beams. The repair materials employed included geopolymer material, and epoxy resin mortar, while carbon fiber sheets were used as reinforcement in select cases. The repair materials were applied to the rectangular and square-grooved specimens, with the carbon fiber sheets attached to the tension side of the specimens. To evaluate the flexural strength of the concrete specimens, a third-point loading test was conducted. The test results indicated that the geopolymer exhibited higher compressive strength and shrinkage rate compared to the epoxy resin mortar. Furthermore, the specimens reinforced with carbon fiber sheets demonstrated even greater strength than the benchmark specimens. In terms of flexural strength under cyclic third-point loading tests, the carbon fiber-reinforced specimens exhibited the ability to withstand over 200 cycles of repeated loading at 0.8 times the ultimate load. In contrast, the benchmark specimens could only withstand seven cycles. These findings highlight that the use of carbon fiber sheets not only enhances compressive strength but also improves resistance to cyclic loading.

## 1. Introduction

Civil engineers are increasingly faced with the challenge of improving the performance of existing structures, aiming to extend their service life by restoring their mechanical strengths and enhancing their strength beyond their original levels. Many of these structures were designed according to regulations and requirements that are no longer up to date, failing to meet the standards of modern buildings. Exposure to natural forces, human activities, or the effects of time can result in cracks, erosion, or damage. Repairing and reinforcing these aging structures presents a more cost-effective approach compared to redevelopment, as it reduces resource consumption and minimizes the environmental impact associated with new construction.

Given that the majority of these buildings do not meet the design requirements stipulated by current regulations, it becomes essential to carry out necessary repairs and reinforcement engineering to ensure the continued use of the building, especially when partial structural damage occurs, without compromising the overall structural integrity. Consequently, retrofitting reinforced concrete structures remains a critical and pressing issue.

Generally, when reinforced concrete (RC) structures experience crack formation due to external forces, reinforcement is typically carried out considering the crack size and location. Often, cracks tend to occur at locations subjected to significant structural loading, such as the maximum bending point in a reinforced concrete beam. Prior to implementing reinforcement measures, thorough observations are conducted to assess the depth of the cracks, determining whether steel repair is necessary prior to material restoration. During the repair process, various methods are employed depending on the specific structural elements involved. Previous research studies have demonstrated the efficacy of utilizing different types of repair materials, such as alkali-activated slurry and high-performance fiber cement, to effectively enhance the flexural strength of the structure [1,2,3,4,5,6,7].

Although the utilization of repair materials for the inclined surface of a concrete beam can improve its flexural strength, the degree of enhancement remains limited. To achieve significant improvements in bending strength, the retrofitting of high-polymer composite materials has been explored [8,9,10,11]. Notably, the application of carbon fiber sheets as reinforcement on the underside of test specimens during flexural tests has demonstrated substantial enhancements in flexural strength [12,13,14,15,16,17,18,19].

To replicate deteriorated concrete slabs, a previous study employed concrete slabs with deliberately introduced notches [20]. By incorporating various opening notches into the test specimens, the simulation aimed to represent the retrofitting process of an old building with a new elevator shaft. Reinforcing the opening area using carbon fiber sheets resulted in flexural strengths surpassing those achieved through traditional reinforcement methods [21,22,23,24,25,26,27]. 

As previously mentioned, the application of carbon fiber sheets has been proven to enhance the flexural strength of specimens. Several studies have indicated that the use of carbon fiber sheets not only improves flexural strength but also enhances impact resistance and fatigue resistance of the specimens [26,28]. Furthermore, the utilization of ultra-thin high early-strength fiber concrete has been shown to enhance the specimens’ ability to withstand cyclic loading [29]. 

Geopolymer material presents a range of benefits in scientific and technical contexts. Notably, it exhibits an environmentally friendly composition that diminishes reliance on natural resources and promotes the recycling of industrial waste. Moreover, geopolymer material possesses a reduced carbon footprint, improved durability, and exceptional versatility, enabling customization to suit particular application needs [30,31,32]. It is worth noting that existing literature primarily concentrates on examining the efficacy of geopolymer materials in repairing cracks [33,34,35,36], while research specifically comparing inclined surface repair methods remains limited.

The objective of this study is to investigate the potential for achieving complete restoration of the original strength in damaged concrete sections by employing geopolymer as a repair material. Specifically, this research focuses on assessing the feasibility of replacing traditional epoxy resin mortar with geopolymer material for repairing inclined surfaces. Furthermore, the research aims to evaluate the performance of retrofitting grooved concrete specimens using carbon fiber sheets. To achieve these goals, the compressive strength and shrinkage of the two repair materials were measured following ASTM C109 [37] and ASTM C490/C490M-21 [38], respectively. Both materials were applied to rectangular and square-grooved reinforced concrete unidirectional slabs. Following the repair process, the specimens were further reinforced with carbon fiber sheets and subjected to third-point loading and cyclic third-point loading tests. The test results were analyzed to compare and discuss the differences between geopolymer material and commercially available epoxy resin mortar. Additionally, the static load-bearing capacity and cyclic load-bearing capacity were compared between specimens with and without the use of carbon fiber sheets.

## 2. Materials

### 2.1. Epoxy Resin Mortar

Epoxy resin is a thermosetting material commonly used as an adhesive and coating. It is most commonly produced by the reaction of epichlorohydrin and phenoxyethanol. In this study, the epoxy resin A and resin B (SB-LEM) were provided by SamBond International Co., Taipei, Taiwan, which were blended in a fixed ratio of 2:1. This particular resin sand mixture exhibits favorable properties, such as lightweight nature and high adhesion. Epoxy resin sand mixtures are commonly utilized for inclined surface repairs due to their excellent weather resistance and ability to mitigate the corrosion impact on steel reinforcement during crack repairs. The resin sand mixture employed in this research is specifically designed for construction joints and overhead repairs, and it adheres to a predefined proportioning scheme.

### 2.2. Geopolymer Material

Geopolymer materials are non-crystalline or semi-crystalline substances commonly derived from raw materials abundant in silicon or aluminum, such as metakaolin, coal fly ash, or ground-granulated blast-furnace slag powder. The production of geopolymer involves mixing the raw materials with an alkaline solution, which reacts with the particle surfaces of the raw materials, dissolving silica gel and aluminum gel [39,40]. This process leads to the formation of a continuous network-like structure composed of aggregated silica gel and aluminum gel. After dewatering and drying, a three-dimensional framework structure is ultimately established. The concept of geopolymer technology was introduced in 1976 [41], and since then, numerous academic studies have investigated this material. Geopolymer exhibits advantages such as simplified production and easy application, and once hardened, its properties resemble those of cement. Consequently, geopolymer was chosen as the repair material for this study and will be compared with other materials in related tests [42,43,44,45]. The geopolymer material employed in this research primarily consisted of ground-granulated blast-furnace slag powder (S4000, CHC Resources Corporation, Kaohsiung, Taiwan) and an alkaline solution (sodium silicate and sodium hydroxide, YuMing Chemical Co., LTD., Taipei, Taiwan) in a weight ratio of 1:0.65 for ground-granulated blast-furnace slag powder to alkaline solution.

### 2.3. Carbon Fiber Sheet

This study used a carbon fiber sheet infused with epoxy resin, which was attached to the overhead surface of the concrete specimens. This material was provided by Sheng Peng Applied Materials Co., Ltd. (Yunlin, Taiwan). The material specifications are shown in Table 1.

## 3. Test Methods

The experimental plan encompassed a comprehensive series of tests, including assessments of material shrinkage, material compressive strength, third-point loading, and cyclic third-point loading. Through the analysis of material shrinkage and compressive strength test results, an optimized design proportion was determined by blending dry sand with the geopolymer material. The aim was to achieve a design proportion that would demonstrate a satisfactory level of compressive strength while exhibiting a minimal shrinkage rate. This specific design proportion was subsequently employed to evaluate the performance of the geopolymer material under third-point loading and cyclic third-point loading conditions.

The nomenclature assigned to the specimens used in the material property tests is as follows: “E” denotes epoxy resin mortar, “G” represents geopolymer material, and “S” indicates the addition of dry sand to the geopolymer material. The specific quantity of dry sand incorporated into the geopolymer material is indicated by the value following the symbol “S”. For instance, if the geopolymer material was blended with 150% dry sand, the corresponding specimen would be labeled as “G-S150”, as shown in Table 2.

### 3.1. Shrinkage Test for the Repair Materials

The shrinkage rate is a critical parameter to consider when evaluating repair materials. In the case of geopolymers, the loss of residual moisture occurs upon contact with the air surface following the complete reaction of alkaline liquid and slag. This phenomenon results in shrinkage. If shrinkage occurs on the underside of the repaired area, it can lead to the formation of cracks between the repair material and the substrate. These cracks may cause the repair material to detach or create pathways for water infiltration, thereby compromising the effectiveness of the repair. To gain insights into the shrinkage behavior of geopolymers and epoxy resin mortar, this experiment was conducted. Following the guidelines outlined in ASTM C490/C490M-21 [38], specimens measuring 2.5 cm × 2.5 cm × 28.5 cm were prepared, and a shrinkage test was performed using a dedicated shrinkage tester.

### 3.2. Compressive Test

Geopolymer materials exhibit cement-like properties, and the repaired surface is typically subjected to tensile stress. It is worth noting that the tensile strength of cement is approximately one-tenth of its compressive strength. To gain a comprehensive understanding of the properties of geopolymer and epoxy resin mortar, compressive tests were conducted in accordance with the guidelines specified in ASTM C109/C109M-21 [37]. The repair materials were cast into 5 × 5 × 5 cm molds and allowed to cure for seven days prior to testing.

To investigate the compressive strength of the geopolymer material with varying proportions of dry sand, additional compressive tests were performed. Specifically, materials incorporating 150% and 200% weight ratios of sand to geopolymer material were prepared. After a curing period of seven days, these specimens were subjected to compressive testing using a universal testing machine, following the standards outlined in ASTM C109/C109M-21 [37]. The compression rate employed during the tests was set at 100,000 N per minute.

### 3.3. Third-Point Loading Test

In this subsection, unidirectional reinforced concrete slabs were specifically designed and subjected to third-point loading tests. The design process entailed creating grooves of varying sizes on the slabs to simulate concrete cracks, which were subsequently repaired. Some of the test specimens were further reinforced by incorporating carbon fiber sheets in addition to the repair materials used to fill the grooves. The study aims to compare the bending strength of all the specimens after the repair and reinforcement processes.

The design of the unidirectional reinforced concrete slabs in this subsection adheres to the guidelines specified in ACI-318-05. Specifically, #3 mesh reinforcing steel was employed and positioned 3 cm above the tensile side of the test specimens. The slabs were cast using a wooden mold, and the concrete utilized had an average strength of 21 MPa.

**Figure 1:**
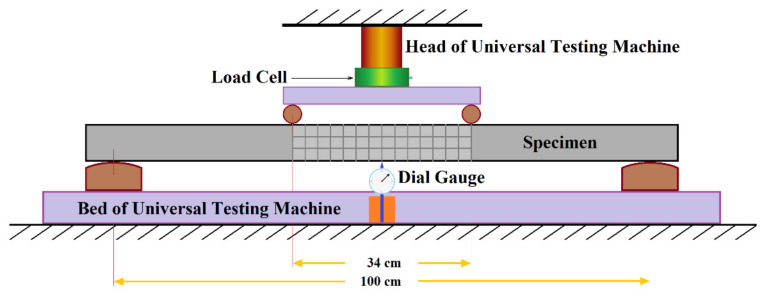
The schematic diagram of the third-point loading test.

Figure 1 illustrates the third-point loading test employed in this study, which was conducted following the specifications outlined in ASTM C78/C78M-22 [46]. However, it is important to note that, as the primary focus of this research centers around concrete slabs rather than the beams referenced in the ASTM standard, test specimens with a total length of 120 cm and a height of 7 cm were utilized. The lower support span was set at 100 cm, with the middle one-third span measuring 34 cm. Furthermore, the study involved subjecting the specimens to cyclic third-point loading under various loads, as depicted in Figure 1.

**Figure 2:**
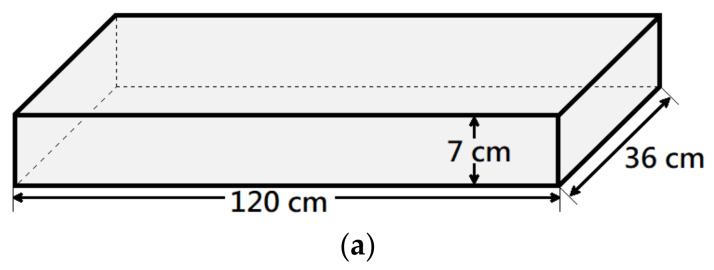

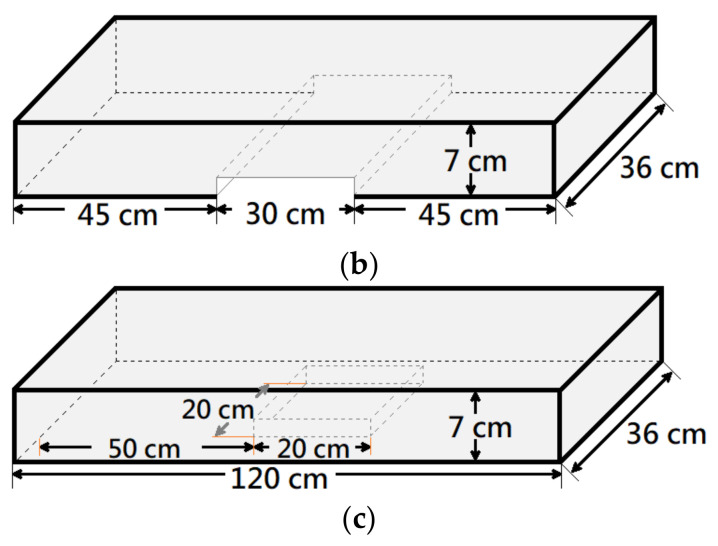
The dimension of specimens: (**a**) benchmark; (**b**) rectangular groove; (**c**) square groove.

This study involved the fabrication of three distinct types of unidirectional reinforced concrete slabs, which were subsequently further categorized. Each unidirectional reinforced concrete slab had dimensions of 120 cm × 36 cm × 7 cm, as shown in Figure 2. The three types of concrete slabs employed in this study were as follows: (1) reinforced concrete (RC) slab without any repair (referred to as B), as seen in Figure 2a; (2) RC slab featuring a 30 cm × 36 cm × 2 cm rectangular groove on the tensile side (referred to as R), as seen in Figure 2b; and (3) RC slab with a 20 cm × 20 cm × 2 cm square groove on the tensile side (referred to as S), as seen in Figure 2c. 

The concrete slabs with grooves, namely the R slabs and S slabs, were further divided into three subgroups: (1) without any repair, (2) repaired with geopolymer (referred to as G), and (3) repaired with epoxy resin mortar (referred to as E). Additionally, within the subgroups involving repair, certain specimens were reinforced with carbon fiber sheets (referred to as C). The nomenclature for the concrete slab specimens can be found in Table 3. It is important to note that each type of specimen listed in Table 3 was represented by two samples, resulting in a total of 22 test samples. Subsequently, third-point loading tests were conducted following a seven-day period after the completion of the repair process.

### 3.4. Cyclic Third-Point Loading Test

The experimental setup and equipment utilized in this study were identical to those employed in the third-point loading test. The objective of this experiment was to subject the test specimens to cyclic third-point loading until they reached their ultimate strength and experienced failure. The ultimate strength of each specimen was then recorded. Subsequently, specimens of the same type were subjected to loading at 0.7, 0.8, and 0.9 times their ultimate strength, and the loading process was repeated until failure occurred. The number of cycles required for failure was documented to assess the fatigue properties of the specimens under cyclic third-point loading.

Each test specimen was divided into ultimate strength categories of 0.7, 0.8, and 0.9 times their ultimate strength. Consequently, each type of specimen consisted of four specimens: one for determining the ultimate loading strength and three for conducting cyclic third-point loading tests. The purpose of these tests was to evaluate the performance of each repair material in reinforced concrete slabs subjected to cyclic third-point loading following repair. The naming convention for the cyclic third-point loading specimens followed a similar format to that of the third-point loading test, with the addition of a prefix “C” to indicate the specimens underwent cyclic loading. Furthermore, a suffix “P” was added after the loading ratio (e.g., 0.7P, 0.8P, or 0.9P) to denote that the specimens were subjected to loading at a ratio of their ultimate strength. For example, the designation “S-E-C-0.9P” referred to a square-grooved specimen repaired with epoxy sand mortar, reinforced with a carbon fiber sheet, and subjected to cyclic third-point loading at 0.9 times its ultimate loading strength.

### 3.5. Specimen Preparation for Third-Point Loading Test

To provide a comprehensive description of the specimen production process for the third-point loading and cyclic third-point loading tests, the fabrication method is categorized into two sections: specimen fabrication and specimen repair and reinforcement. This division allows for a detailed explanation of the procedures involved in each stage.

#### 3.5.1. Specimen Fabrication

**Figure 3:**
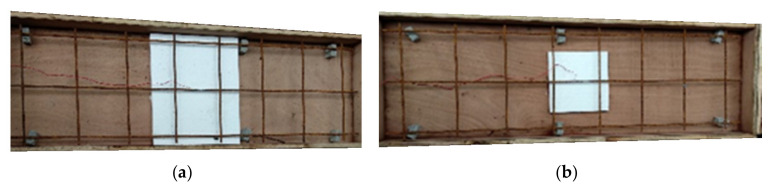
Complete fabrication of wooden molds with polystyrene boards for the rectangular and square-grooved specimens: (**a**) wooden molds with polystyrene boards for the rectangular-grooved specimens; (**b**) wooden molds with polystyrene boards for the square-grooved specimens.

The fabrication process of a unidirectional reinforced concrete specimen involved the assembly of a wooden mold on-site, followed by the sequential placement of polystyrene boards to create the desired groove size. Subsequently, #3 steel mesh was positioned within the mold before pouring the concrete, as depicted in Figure 3. To measure the ultimate strain of the specimen during loading, strain gauges were affixed to the steel mesh corresponding to the tensile side of the specimen.

The assembled mold was placed on a flat surface for casting. To maintain the strength stability for each reinforced concrete specimen, on-site concrete quality control tests, including chlorides test and slump test, were performed in conjunction with quality control personnel during casting. To maintain a flat surface of the specimens, tamping, compaction tools, and trowels were used for compaction and smoothing.

After the concrete casting was completed, a curing method was performed, and after 21 days of solidification, repair materials were cast, and reinforcement materials were attached. The compressive strength of the concrete cylindrical specimens was performed on the same day as the third-point loading test for each group of specimens to confirm the quality of concrete in each test.

#### 3.5.2. Repair and Reinforcement Method Third-Point Loading Test

**Figure 4:**
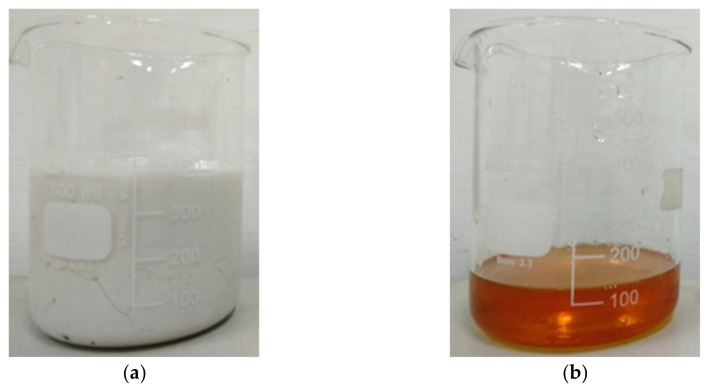
Primer resin: (**a**) resin A; (**b**) resin B.

Before applying the repair material, a primer resin (EP 220) provided by Kuosen Enterprise Co., Ltd., Taoyuan, Taiwan, was used as the bonding material. A primer resin adhesive agent was used for each repair specimen before repairing with the repair material. Figure 4 shows the primer resin A and resin B. The composition of the primer resin consists of resin A and resin B, which were blended in a fixed ratio of 4:1. After blending, the mixture was applied to the grooved area of the specimen to be repaired using a brush and allowed to sit for 15 min before repairing with the repair material. 

**Figure 5:**
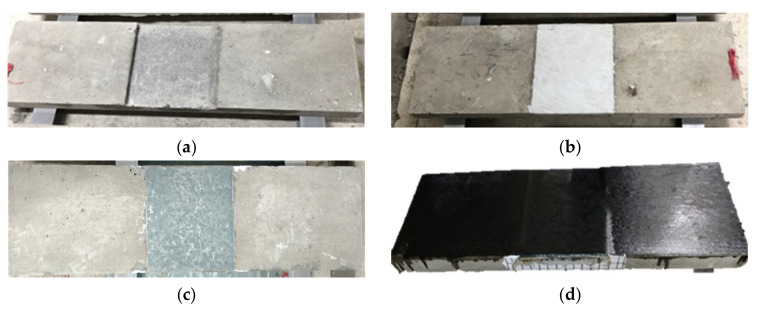
Rectangular-grooved specimens after repair and reinforcement: (**a**) specimen R; (**b**) specimen R-E; (**c**) specimen R-G; (**d**) specimen R-G-C and specimen R-E-C.

**Figure 6:**
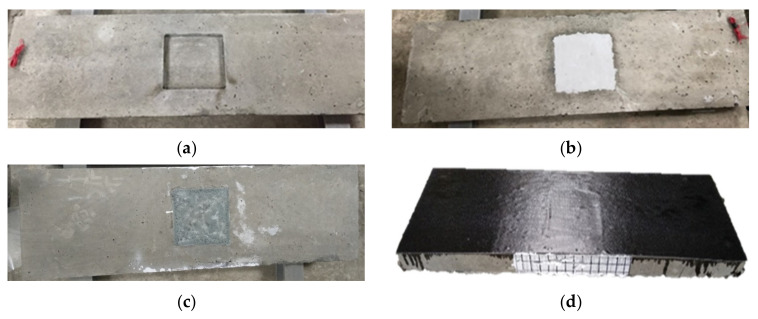
Square-grooved specimens after repair and reinforcement: (**a**) specimen S; (**b**) specimen S-E; (**c**) specimen S-G; (**d**) specimen S-G-C and specimen S-E-C.

Figure 5 and Figure 6 are the completed images of R-type (rectangular groove) and S-type (square groove) specimens, respectively, after repair and reinforcement. 

## 4. Results of the Repair Material Properties

This section is divided into compressive tests and shrinkage tests of repair materials. The ratio of geopolymer repair material used in the later third-point loading test was determined by the results of this section.

### 4.1. Shrinkage Measurement Result

The test results were recorded in accordance with the guidelines outlined in ASTM C490/C490M-21 [31]. Following solidification, all specimens were carefully demolded, and their lengths were measured to be 28.5 cm. Each group of specimens consisted of three individual samples, and each sample was measured three times to ensure accuracy, with the average value being recorded.

The summarized average values of the shrinkage test results for all repair materials are presented in Table 4. Notably, the geopolymer specimen (G) without any sand mixing exhibited the highest average shrinkage of 1.305% among all specimens. On the other hand, the geopolymer specimen (G-S150) mixed with sand at a weight ratio of 150% displayed an average shrinkage of 0.736%. The geopolymer specimen (G-S200) mixed with sand at a weight ratio of 200% exhibited the third lowest shrinkage rate, with an average shrinkage of 0.297%, which was the lowest among all geopolymer specimens. It is worth mentioning that the epoxy resin mortar specimen (E) demonstrated the lowest shrinkage rate among all tested materials, with a mere 0.015% shrinkage rate.

The results obtained from the shrinkage test, as presented in Table 4, revealed notable differences in the shrinkage behavior between the geopolymer specimens and the epoxy resin mortar. The geopolymer specimens exhibited higher levels of shrinkage compared to the epoxy resin mortar. However, incorporating sand into the geopolymer material proved to be an effective strategy for reducing shrinkage. Specifically, when the geopolymer material was mixed with a sand weight ratio of 200% (G-S200), the shrinkage rate decreased significantly from 1.305% in specimen G to 0.297% in specimen G-S200. In contrast, the epoxy resin mortar demonstrated the lowest shrinkage rate among all the tested repair materials. Thus, the ranking of shrinkage rates, from highest to lowest, can be summarized as follows: G > G-S150 > G-S200 > E.

### 4.2. Compressive Test Results 

The compressive strength of the repair materials was determined through compressive tests conducted in accordance with ASTM C109/C109M-21 [30]. The average compressive strength of all repair materials after seven days is presented in Table 5. Notably, specimen G-S150 exhibited the highest average compressive strength, while specimen E demonstrated the lowest strength among the tested materials. It is noteworthy that an increase in the dry sand content in the geopolymer material appeared to enhance its compressive strength, as evidenced by the comparison between specimens G and G-S150. However, it is important to mention that there seemed to be a maximum threshold, as further additions of dry sand beyond this point resulted in a decrease in compressive strength. This observation is supported by the values obtained for specimens G-S150 and G-S200.

The results of the compressive strength test, as presented in Table 5, demonstrated that geopolymer specimens exhibited higher compressive strength compared to epoxy resin mortar, with a strength increase ranging from 284% to 387%. Among them, specimen G-S150, which consisted of geopolymer material mixed with a weight ratio of 150% dry sand, exhibited the highest compressive strength at 73.78 MPa. The compressive strength ranked from highest to lowest was G-S150 > G-S200 > G > E.

To evaluate the effectiveness of geopolymer and carbon fiber sheets for repairing surfaces at inclined positions, specimen G-S150 was selected as the repair material for the third-point loading and cyclic third-point loading tests. This decision was based on its high compressive strength and low shrinkage rate within the geopolymer material category. Consequently, all subsequent geopolymer material repaired specimens incorporated a weight proportion of 150% dry sand. For example, the specimen “R-G-C” denoted a rectangular-grooved specimen repaired with geopolymer material mixed with a weight proportion of 150% dry sand and reinforced with a carbon fiber sheet.

To compare the feasibility of repairing damaged surfaces using geopolymer, this research employed epoxy resin mortar as the control group and geopolymer as the experimental group. Both groups were reinforced with carbon fiber sheets. The repair process primarily involved the application of geopolymer and epoxy resin mortar, respectively, while the carbon fiber sheets provided additional reinforcement.

## 5. Third-Point Loading Test Results 

This section classified the specimens into two distinct sets: one comprising specimens with rectangular grooves (designated as specimen set R) and the other consisting of specimens with square grooves (designated as specimen set S). Each set comprised two individual specimens, upon which third-point loading tests were conducted. The resulting test outcomes, including failure modes, were meticulously documented for analysis and evaluation.

### 5.1. Rectangular-Grooved Specimens

Table 6 shows the result for the ultimate load, displacement, and their corresponding average values of the benchmark and the specimens with rectangular grooves. 

**Figure 7:**
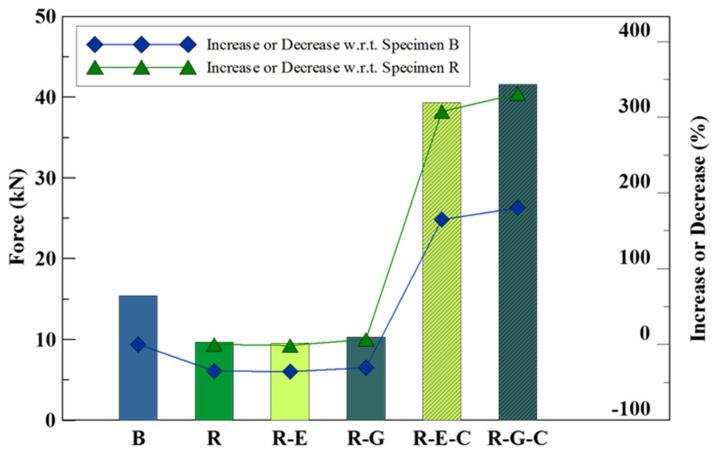
Average ultimate loading of the benchmark and rectangular-grooved specimens.

The average flexural strength of specimens with rectangular grooves was plotted in the bar chart in Figure 7.

From the analysis of Figure 7, it is apparent that undamaged concrete slab specimens (designated as Bs) exhibited higher ultimate loading strength compared to damaged concrete slab specimens (designated as Rs), which simulated the presence of cracks. The impact of repairing the damaged specimens with geopolymer material mixed with dry sand (R-Gs) and epoxy resin mortar (R-Es) on their ultimate loading strength was minimal, with limited observable improvements that could potentially be attributed to experimental errors. However, notable enhancements were observed when the repaired specimens were additionally reinforced with a carbon fiber sheet. The combination of geopolymer material or epoxy resin mortar with carbon fiber reinforcement resulted in a substantial increase in flexural strength for the repaired specimens, ranging from 300% to 330% in comparison to the unrepaired specimen Rs. Moreover, the flexural strength of the reinforced repaired specimens surpassed that of the undamaged concrete slab specimen B by 165% to 180%. Detailed visual documentation of the rectangular-grooved specimens at the point of failure, which occurred at different loading values during the third-point loading test, can be found in Table 7.

The examination of Table 7 reveals distinct patterns in the failure modes of the specimens. Specimen R-E, representing the epoxy resin mortar repair, exhibited cracks at both edges of the grooved section. Conversely, specimen R-G, which underwent repair using geopolymer material, displayed cracks at the center as well as both edges of the grooved section. The presence of cracks in both the geopolymer material and the concrete slab suggests that the geopolymer material possesses material properties similar to those of concrete, as it experienced cracking in a manner consistent with the surrounding substrate.

### 5.2. Square-Grooved Specimens

Table 8 shows the results for the ultimate load, displacement, and respective average values of the benchmark specimens and the specimens with square grooves.

**Figure 8:**
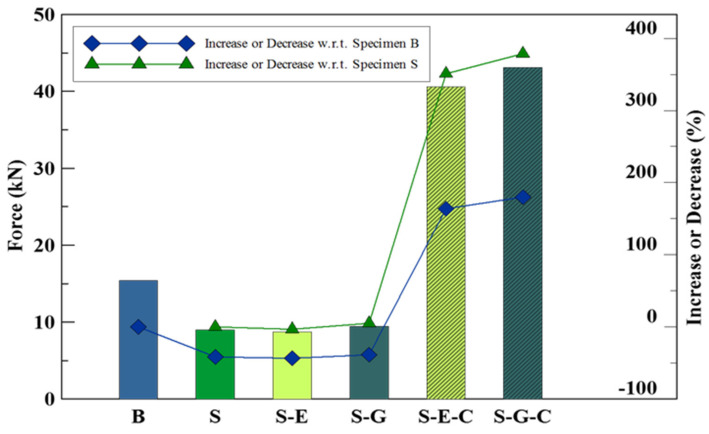
Average ultimate bearing capacity of the benchmark and specimens with square grooves.

Figure 8 is the bar chart of the average flexural strength of specimen set S, created from Table 8.

In this subsection involving square-grooved specimens, the results of the third-point loading test exhibited similarities to those observed in the previous subsection. The repair of damaged concrete slab specimens (S) using either epoxy resin mortar or geopolymer material mixed with 150% dry sand had a limited effect on enhancing their flexural strength, resulting in less than a 5% improvement. However, the introduction of carbon fiber sheet reinforcement to the repaired specimens yielded significant enhancements. Irrespective of the repair material utilized, the flexural strength of the carbon fiber sheet-reinforced specimens exhibited a remarkable increase of 350% to 380% compared to the unrepaired specimens. Furthermore, the flexural strength of the carbon fiber sheet-reinforced repaired specimens surpassed that of the undamaged concrete slab (specimen B) by 160% to 180%.

## 6. Cyclic Third-Point Loading Test Results

This section presents the findings of the cyclic third-point loading tests, categorized into two groups: rectangular-grooved specimens and square-grooved specimens. Prior to conducting the cyclic third-point loading test, one cylindrical specimen from each group underwent a compression test to ensure consistent concrete quality throughout the experiment. Subsequently, another specimen from each group was subjected to a flexural test to determine the ultimate flexural strength of the respective group. The remaining specimens in each group were then subjected to cyclic loading tests at varying ratios of their ultimate flexural strength.

### 6.1. Rectangular-Grooved Specimens

**Figure 9:**
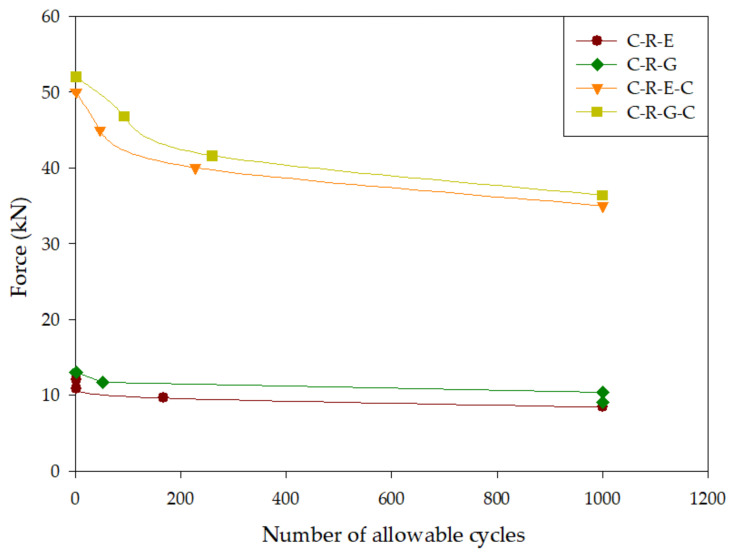
Fatigue curve of rectangular-grooved specimens during cyclic third-point loading test.

Table 9 presents the outcomes of the cyclic third-point loading test conducted on rectangular-grooved specimens. In the table, the notation “N” indicates specimens that experienced no failure, while “Y” signifies specimens that failed. Analysis of Table 9 reveals that, across the four distinct repair methods employed on the specimens, they were able to endure more than 1000 cycles without failure when subjected to cyclic third-point loading tests at an ultimate load ratio of 0.7. Furthermore, under cyclic third-point loading tests at an ultimate load ratio of 0.8, specimen C-R-G, repaired using geopolymer material, demonstrated a capacity to withstand more than 1000 cycles without failure. Notably, under cyclic third-point loading tests at an ultimate load ratio of 0.9, specimen C-R-G-C, which underwent repair with geopolymer material and reinforcement with carbon fiber patches, exhibited the highest cycle count. Detailed information regarding the ultimate load percentages and the corresponding cyclic third-point loading test cycles for each specimen can be found in Table 9, while Figure 9 depicts these data as an S-N curve.

Figure 9 depicts the correlation between the number of load cycles-to-failure and the rectangular-grooved specimens in the cyclic third-point loading test. For instance, let us consider specimen C-R-G-C, which exhibited an ultimate load of 52 kN. At 0.9 times the ultimate load, the load amounted to 46.8 kN, while at 0.8 times and 0.7 times the ultimate load, the loads were 41.6 kN and 36.4 kN, respectively.

The findings presented in Figure 9 clearly indicate that the incorporation of carbon fiber sheet reinforcement enhances the fatigue resistance of the specimens. Notably, specimen C-R-G-C demonstrated the highest fatigue resistance among the specimens examined. Even under the cyclic third-point loading test at 0.9 times the ultimate load, it exhibited the greatest fatigue load capacity when compared to the other rectangular specimens.

### 6.2. Square-Grooved Specimens

Table 10 presents the results obtained from the cyclic third-point loading test conducted on square specimens. The table distinguishes between failure, indicated by “Y”, and no failure, indicated by “N”. Upon analyzing Table 10, it becomes apparent that, during the cyclic third-point loading test at 0.7 times the ultimate load, all repaired specimens were capable of withstanding more than 1000 repetitions without experiencing failure. Furthermore, at 0.8 times the ultimate load, both specimens repaired with geopolymer materials (C-S-G) and epoxy mortar (C-S-E) exhibited a remarkable ability to endure over 1000 repetitions without failure.

**Figure 10:**
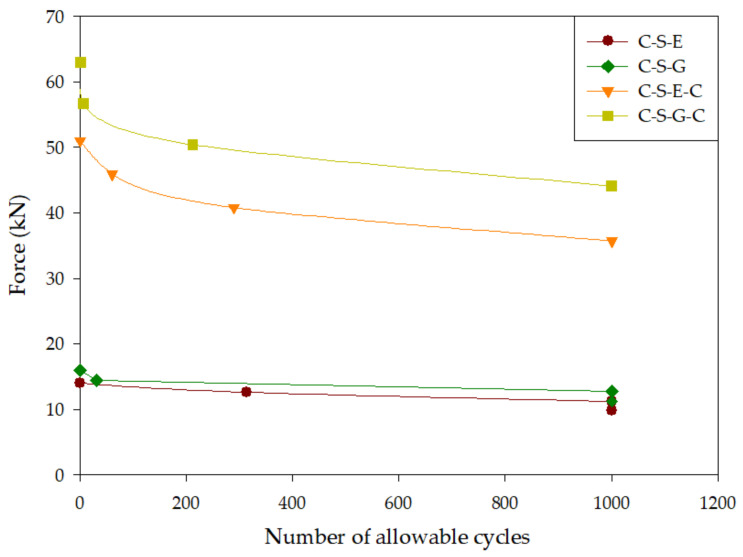
Fatigue curve of square-grooved specimens during cyclic third-point loading test.

Figure 10 provides a visual representation of the data and illustrates that the utilization of carbon fiber reinforcement enhances the fatigue resistance of the specimens. Notably, specimen C-S-G-C demonstrated the highest fatigue resistance under cyclic third-point loading. At 0.8 times its ultimate load, the load strength of this specimen approached the ultimate strength observed in specimen C-S-E-C. It is worth mentioning that the cyclic third-point loading fatigue resistance of specimen C-S-G-C consistently surpassed that of specimen C-S-E-C.

The findings derived from the cyclic third-point loading test on square-grooved specimens clearly demonstrate that the incorporation of carbon fiber sheet reinforcement enhances the fatigue resistance of specimens under tensile stress. Prior to the application of carbon fiber sheet reinforcement, the square-grooved specimen could only withstand 32 repeated loads at 14.4 kN. However, after reinforcement, the specimen exhibited the ability to endure over 1000 repeated loads at 40 kN.

The fatigue resistance of specimens repaired with geopolymer material during the cyclic third-point loading test surpassed that of specimens repaired with epoxy resin mortar. This observation aligns with the outcomes of the third-point loading test, which revealed that specimens repaired with geopolymer material exhibited greater strength compared to those repaired with epoxy resin mortar. A similar trend was also evident in the results of the cyclic third-point loading test conducted using carbon fiber reinforcement.

Despite a slightly higher shrinkage rate, the inclusion of geopolymer material with dry sand proves to be a viable alternative to epoxy resin mortar as a repair material. This alternative possesses superior material strength and leads to increased compressive strength in the concrete slab.

## 7. Conclusions

The objective of this study was to investigate the viability of utilizing geopolymer materials for the repair of reinforced concrete beams. The research yielded the following conclusions:

1. The incorporation of sand in geopolymer materials leads to a decrease in shrinkage. Specifically, the shrinkage values decreased from 3.718 mm to 0.845 mm as the amount of sand increased.
2. The addition of sand to geopolymer materials can enhance their compressive strength; however, there exists a threshold beyond which excessive sand content can result in a decline in strength.
3. The findings obtained from the third-point loading test indicate that the repair of damaged concrete slabs using either epoxy resin mortar or geopolymer materials alone yields only marginal improvements in flexural strength. Moreover, the original undamaged strength of the slabs cannot be fully restored through these repair methods.
4. When repaired concrete slabs are reinforced with carbon fiber sheets, their flexural strength can increase by 160% to 180% in comparison to undamaged concrete slabs.
5. The results of the cyclic third-point loading test clearly demonstrate that the utilization of carbon fiber sheet reinforcement significantly enhances the fatigue resistance of concrete slabs.
6. Based on the aforementioned conclusions, it is recommended to both repair and reinforce damaged concrete slabs to extend their service life. Repairing primarily contributes to restoring the shape of the slabs, while reinforcement elevates their flexural strength, surpassing even the pre-damaged state.

## Figures and Tables

**Table 1:** Specification of the carbon fiber sheets.

Property (Unit)	Value
Fiber area weight (g/m^2^)	300
Elastic modulus (GPa)	253
Ultimate strain at failure	0.018
Tensile strength at failure (MPa)	4020.3
Thickness (mm/layer)	0.160

**Table 2:** Abbreviation of the repair material.

Abbreviation	Description
G	Geopolymer material
G-S150	Geopolymer mixed with 150% dry sand
G-S200	Geopolymer mixed with 200% dry sand
E	Epoxy resin mortar

**Table 3:** Naming of the specimens in the third-point loading test.

Specimen	Description
B	RC slab without groove and repair (Benchmark)
R	RC slab with rectangular groove and without repair
R-E	RC slab with rectangular groove repaired with epoxy resin mortar
R-E-C	RC slab with rectangular groove repaired with epoxy resin mortar and reinforced with carbon fiber sheet
R-G	RC slab with rectangular groove repaired with geopolymer material
R-G-C	RC slab with rectangular groove repaired with geopolymer material and reinforced with carbon fiber sheet
S	RC slab with square groove and without repair
S-E	RC slab with square groove repaired with epoxy resin mortar
S-E-C	RC slab with square groove repaired with epoxy resin mortar and reinforced with carbon fiber sheet
S-G	RC slab with square groove repaired with geopolymer material
S-G-C	RC slab with square groove repaired with geopolymer material and reinforced with carbon fiber sheet

**Table 4:** Average shrinkage rate of the repair materials.

Specimen	Shrinkage (mm)			Average Shrinkage (mm)	Average Shrinkage Rate (%)
G	3.721	3.701	3.735	3.718	1.305
G-S150	2.242	2.118	2.247	2.202	0.736
G-S200	0.867	0.835	0.832	0.845	0.297
E	0.038	0.043	0.036	0.039	0.015

**Table 5:** Average compressive strength of the repair materials (at seven days).

Specimen	Compressive Strength (MPa)			Average Compressive Strength (MPa)
G	60.19	64.70	61.80	62.23
G-S150	70.92	76.51	73.91	73.78
G-S200	65.01	69.19	66.20	66.80
E	19.08	23.52	22.97	21.85

**Table 6:** Ultimate flexural strength for the benchmark and the rectangular-grooved specimens.

Specimen	Ultimate Load (kN)	Displacement (mm)	Average Ultimate Load (kN)	Average Displacement (mm)
B-01	15.3	15.10	14.8	16.1
B-02	14.4	17.10		
R-01	9.4	23.80	9.6	25.2
R-02	9.8	26.69		
R-E-01	9.8	24.74	9.5	21.5
R-E-02	9.3	18.39		
R-G-01	10.2	21.07	10.2	21.1
R-G-02	10.3	21.27		
R-E-C-01	40.2	16.60	39.3	16.7
R-E-C-02	38.3	16.84		
R-G-C-01	40.7	18.43	41.9	18.4
R-G-C-02	43.2	18.32		

**Table 7:** Photos of rectangular-grooved specimens at failure in third-point loading tests.

Specimen	Destroy Photo	Directions
B	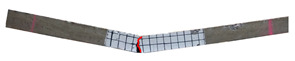	Final failure occurs after flexural cracks appear in the middle.
R	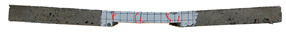	Due to the smaller moment of inertia at the grooved section, cracking occurs at the grooved section during failure.
R-E	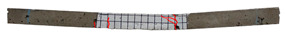	Cracks occur at the grooved section and the repair material of the reinforced concrete; hence failure occurs at the grooved section.
R-G	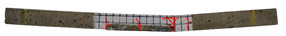	After flexural cracks appear in the center, cracks occur at the interface between the repair material and the specimen, resulting in final failure.
R-E-C	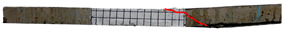	After delamination of the carbon fiber sheet, flexural-shear cracks appear in the concrete and cause failure.
R-G-C	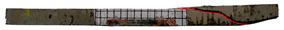	After delamination of the carbon fiber sheet, flexural-shear cracks appear in the concrete and cause failure.

**Table 8:** Ultimate flexural strength for the benchmark and the square-grooved specimens.

Specimen	Ultimate Load (kN)	Displacement (mm)	Average	
			Ultimate Load (kN)	Displacement (mm)
B-01	15.3	15.10	14.8	16.1
B-02	14.4	17.10		
S-01	8.9	19.64	9.0	19.8
S-02	9.1	20.14		
S-E-01	8.3	21.16	8.7	21.8
S-E-02	9.1	22.47		
S-G-01	9.1	21.54	9.45	20.9
S-G-02	9.8	20.45		
S-E-C-01	40.1	20.26	40.6	18.5
S-E-C-02	41.2	16.75		
S-G-C-01	44.6	17.09	43.1	16.9
S-G-C-02	41.6	16.68		

**Table 9:** Test result of cyclic third-point loading of rectangular-grooved specimens.

Name of Specimens	Ultimate Load, P (kN)	0.9 P		0.8 P		0.7 P	
		Force (kN)	Count	Force (kN)	Count	Force (kN)	Count
C-R-E	12	10.8	2	9.6	167	8.4	>1000
C-R-G	13	11.7	51	10.4	>1000	9.1	>1000
C-R-E-C	50	45	46	40	227	35	>1000
C-R-G-C	52	46.8	92	41.6	259	36.4	>1000

**Table 10:** Test result of cyclic third-point loading of square-grooved specimens.

Name of Specimens	Ultimate Load, P (kN)	0.9 P		0.8 P		0.7 P	
		Force (kN)	Count	Force (kN)	Count	Force (kN)	Count
C-S-E	14	12.6	313	11.2	>1000	9.8	>1000
C-S-G	16	14.4	32	12.8	>1000	11.2	>1000
C-S-E-C	51	45.9	61	40.8	289	35.7	>1000
C-S-G-C	63	56.7	7	50.4	212	44.1	>1000

## Data Availability

Not applicable.

## References

[1] Al-Salloum A. (2007). Flexural performance of RC beams repaired with commercial repair materials. J. King Saud Univ. Eng. Sci..

[2] Ahmad S., Elahi A., Barbhuiya S.A., Farid Y. (2012). Use of polymer modified mortar in controlling cracks in reinforced concrete beams. Constr. Build. Mater..

[3] Wang B., Xu S., Liu F. (2016). Evaluation of tensile bonding strength between UHTCC repair materials and concrete substrate. Constr. Build. Mater..

[4] Hor Y., Wee T., Kazutaka S. (2017). Experimental investigation on the behaviour of reinforced concrete slabs strengthened with ultra-high performance concrete. Constr. Build. Mater..

[5] Geraldo R., Teixeira O., Matos S., Silva F., Gonçalves J., Camarini G. (2015). Study of alkali-activated mortar used as conventional repair in reinforced concrete. Constr. Build. Mater..

[6] Al-Majidi H., Lampropoulos P., Cundy B., Tsioulou T., Al-Rekabi S. (2018). A novel corrosion resistant repair technique for existing reinforced concrete (RC) elements using polyvinyl alcohol fibre reinforced geopolymer concrete (PVAFRGC). Constr. Build. Mater..

[7] Loreto G., Leardini L., Arboleda D., Nanni A. (2014). Performance of RC slab-type elements strengthened with fabric-reinforced cementitious-matrix composites. J. Compos. Constr..

[8] Contamine R., Si Larbi A. (2015). Development of a textile reinforced concrete (TRC) to retrofit reinforced concrete structures. Eur. J. Environ. Civ. Eng..

[9] Ebead U., Shrestha K.C., Afzal M.S., Refai A.E., Nanni A. (2017). Effectiveness of fabric-reinforced cementitious matrix in strengthening reinforced concrete beams. J. Compos. Constr..

[10] Radnić J., Matešan D., Grgić N., Baloević G. (2015). Impact testing of RC slabs strengthened with CFRP strips. Compos. Struct..

[11] Si Larbi A., Agbossou A., Hamelin P. (2013). Experimental and numerical investigations about textile-reinforced concrete and hybrid solutions for repairing and/or strengthening reinforced concrete beams. Compos. Struct..

[12] Al-Rousan R., Issa M., Shabila H. (2012). Performance of reinforced concrete slabs strengthened with different types and configurations of CFRP. Compos. Part B Eng..

[13] Hawileh A., Rasheed A., Abdalla A., Al-Tamimi K. (2014). Behavior of reinforced concrete beams strengthened with externally bonded hybrid fiber reinforced polymer systems. Mater. Des..

[14] Al-Sulayvani B.J., Al-Talabani D.N. (2015). Strengthening and repair of circular RC slabs with openings using CFRP strips under repeated loading. Constr. Build. Mater..

[15] Gherdaoui M., Guenfoud M., Madi R. (2018). Punching behavior of strengthened and repaired RC slabs with CFRP. Constr. Build. Mater..

[16] Kim H.-Y., You Y.-J., Ryu G.-S. (2022). Flexural strengthening of RC slabs with lap-spliced carbon textile grids and cementitious grout. Materials.

[17] Mansur de Castro Silva R., de Andrade Silva F. (2020). Carbon textile reinforced concrete: Materials and structural analysis. Mater. Struct..

[18] Awani O., El-Maaddawy T., Ismail N. (2017). Fabric-reinforced cementitious matrix: A promising strengthening technique for concrete structures. Constr. Build. Mater..

[19] Rossi E., Randl N., Mészöly T., Harsányi P. (2021). Flexural strengthening with fiber-/textile-reinforced concrete. ACI Struct. J..

[20] De Domenico D., Urso S., Borsellino C., Spinella N., Recupero A. (2020). Bond behavior and ultimate capacity of notched concrete beams with externally-bonded FRP and PBO-FRCM systems under different environmental conditions. Constr. Build. Mater..

[21] Enochsson O., Lundqvist J., Täljsten B., Rusinowski P., Olofsson T. (2007). CFRP strengthened openings in two-way concrete slabs – An experimental and numerical study. Constr. Build. Mater..

[22] Smith S.T., Kim S.J. (2009). Strengthening of one-way spanning RC slabs with cutouts using FRP composites. Constr. Build. Mater..

[23] Anil Ö., Kaya N., Arslan O. (2013). Strengthening of one way RC slab with opening using CFRP strips. Constr. Build. Mater..

[24] Nahum L., Peled A., Gal E. (2020). The flexural performance of structural concrete beams reinforced with carbon textile fabrics. Compos. Struct..

[25] Schladitz F., Frenzel M., Ehlig D., Curbach M. (2012). Bending load capacity of reinforced concrete slabs strengthened with textile reinforced concrete. Eng. Struct..

[26] Kim H.-Y., You Y.-J., Ryu G.-S., Koh K.-T., Ahn G.-H., Kang S.-H. (2020). Flexural strengthening of concrete slab-type elements with textile reinforced concrete. Materials.

[27] Babaeidarabad S., Loreto G., Nanni A. (2014). Flexural strengthening of RC beams with an externally bonded fabric-reinforced cementitious matrix. J. Compos. Constr..

[28] Rabinovitch O., Frostig Y. (2003). Experiments and analytical comparison of RC beams strengthened with CFRP composites. Compsites Part B Eng..

[29] Ramachandra Murthy A., Karihaloo B., Vindhya Rani P., Shanmuga Priya D. (2018). Fatigue behaviour of damaged RC beams strengthened with ultra high performance fibre reinforced concrete. Int. J. Fatigue.

[30] Růžek V., Dostayeva A.M., Walter J., Grab T., Korniejenko K. (2023). Carbon fiber-reinforced geopolymer composites: A review. Fibers.

[31] Wang T., Fan X., Gao C., Qu C., Liu J., Yu G. (2023). The influence of fiber on the mechanical properties of geopolymer concrete: A review. Polymers.

[32] Pham K.V.A., Nguyen T.K., Le T.A., Han S.W., Lee G., Lee K. (2019). Assessment of performance of fiber reinforced geopolymer composites by experiment and simulation analysis. Appl. Sci..

[33] Zheng C., Mao Z., Chen L., Qian H., Wang J. (2023). Development of a novel rapid repairing agent for concrete based on GFRP waste powder/CGBS geopolymer mortars. J. Build. Eng..

[34] Wang Y.S., Peng K.D., Alrefaei Y., Dai J.G. (2021). The bond between geopolymer repair mortars and OPC concrete substance: Strength and microscopic interactions. Cem. Concr. Compos..

[35] Ding Y.C., Cheng T.W., Dai Y.S. (2017). Application of geopolymer paste for concrete repair. Struct. Concr..

[36] Ding Y.C., Chang W.H., Lee W.H., Wang S., Cheng T.W. (2018). Synthesis of fly ash/metakaolin geopolymer for concrete retrofitting application. Struct. Concr..

[37] (2021). Standard Test Method for Compressive Strength of Hydraulic Cement Mortars (Using 2-in. or [50 mm] Cube Specimens).

[38] (2021). Standard Practice for Use of Apparatus for the Determination of Length Change of Hardened Cement Paste, Mortar, and Concrete.

[39] Lahoti M., Tan K.H., Yang E.H. (2019). A critical review of geopolymer properties for structural fire-resistance applications. Constr. Build. Mater..

[40] Cong P., Cheng Y. (2021). Advances in geopolymer materials: A comprehensive review. J. Traffic Transp. Eng..

[41] Davidovits J. Solid phase synthesis of mineral blockpolymer by low temperature polycondensation of alumino-silicate polymers. Proceedings of the IUPAC Symposium on Long-Term Properties of Polymers and Polymeric Materials.

[42] Liew Y.M., Heah C.Y., Mohd Mustafa A.B., Kamarudin H. (2016). Structure and properties of clay-based geopolymer cements: A review. Prog. Mater. Sci..

[43] Aliques-Granero J., Tognonvi M.T., Tagnit-Hamou A. (2019). Durability study of AAMs: Sulfate attack resistance. Constr. Build. Mater..

[44] Shill S.K., Al-Deen S., Ashraf M., Hutchison W. (2020). Resistance of fly ash based geopolymer mortar to both chemicals and high thermal cycles simultaneously. Constr. Build. Mater..

[45] Ding Y., Shi C.J., Li N. (2018). Fracture properties of slag/fly ash-based geopolymer concrete cured in ambient temperature. Constr. Build. Mater..

[46] (2022). Standard Test Method for Flexural Strength of Concrete (Using Simple Beam with Third-Point Loading).

